# Brain oscillation-synchronized stimulation for major depression: a randomized controlled trial comparing EEG-triggered repetitive TMS with standard iTBS (Acronym: BOSSFRONT2)

**DOI:** 10.1007/s00406-025-02176-9

**Published:** 2026-01-21

**Authors:** Anne Lieb, Brigitte Zrenner, Julia Becker-Sadzio, Pedro Caldana Gordon, Gábor Kozák, Christoph Zrenner, Peter Martus, Ulf Ziemann, Andreas Fallgatter

**Affiliations:** 1 Department of Neurology and Stroke, University of Tübingen, Tübingen, Germany; 2 Hertie Institute for Clinical Brain Research, University of Tübingen, Tübingen, Germany; 3 Temerty Centre for Therapeutic Brain Intervention, Centre for Addiction and Mental Health, Toronto, ON Canada; 4 Department of Psychiatry, University of Toronto, Toronto, ON Canada; 5 Campbell Family Mental Health Research Institute, Centre for Addiction and Mental Health, Toronto, ON Canada; 6 Department of Psychiatry and Psychotherapy, University of Tübingen, Tübingen, Germany; 7 Partner Site Tübingen, German Center for Mental Health (DZPG), University of Tübingen, Tübingen, Germany; 8 Institute for Biomedical Engineering, University of Toronto, Toronto, ON Canada; 9 Institute of Clinical Epidemiology and Applied Biostatistics, University of Tübingen, Tübingen, Germany

**Keywords:** Major depressive disorder, Transcranial magnetic stimulation (TMS), Brain oscillation, Brain state-dependent stimulation, Theta rhythm, Personalized treatment

## Abstract

**Background:**

Major depressive disorder (MDD) is a common severe mental disorder with enormous socioeconomic costs for the patient and society alike. Current pharmacological and psychotherapeutic treatments are ineffective in a substantial fraction of patients and can be accompanied by unwanted side effects.

**Methods:**

Using a novel non-invasive brain stimulation method to specifically target and modulate dysfunctional brain oscillations with high spatial and temporal precision this study will investigate the efficacy of EEG-triggered transcranial magnetic stimulation (TMS) to alleviate depressive symptoms in 28 patients with MDD in a 1:1-randomized, double blind, standard treatment-controlled pilot trial. In the experimental condition, stimulation will be brain oscillation-synchronized based on the EEG-extracted endogenous theta oscillation over left dorsomedial prefrontal cortex (DMPFC). The control condition will consist of standard TMS therapy following an FDA-approved intermittent theta-burst stimulation (iTBS) protocol over left dorsolateral prefrontal cortex (DLPFC). The treatment will be performed over four consecutive weeks (20 sessions) and the primary outcome will be the change in the Montgomery-Åsberg Depression Rating Scale (MADRS) after the last versus before the first treatment session.

**Discussion:**

The aim of the study is to gain exploratory evidence for the feasibility and efficacy of EEG-synchronized TBS of left DMPFC compared to a standard FDA-approved treatment (iTBS of left DLPFC) in a double-blind randomized parallel-design pilot clinical trial. Positive results will pave the way for a larger RCT to prove the superiority of personalized, brain oscillation-synchronized non-invasive brain stimulation therapies as novel, effective and well-tolerated treatment in MDD.

**Trial registration:**

*Trial Registration*: NCT06345651, date of registration: 2024-01-08, https://clinicaltrials.gov/study/NCT06345651The trial is conducted in accord with protocol version number 2022-09, Version 3. Recruitment started in January 2024, and recruitment is ongoing. The recruitment phase is projected to conclude by December 2026.

**Supplementary Information:**

The online version contains supplementary material available at 10.1007/s00406-025-02176-9.

## Introduction

### Background and rationale

Major depressive disorder (MDD) is a prevalent and severe psychiatric illness with a lifetime prevalence of 8–16% [1]. Standard treatments for MDD include antidepressant pharmacotherapy and psychotherapy, which are effective for many patients. However, a substantial fraction of patients does not achieve adequate response with these first-line approaches [2–4]. Such treatment-resistant depression is common—on the order of 20–30% of patients—underscoring the need for alternative or adjunctive interventions. In recent years, several novel therapies have emerged (e.g. ketamine/esketamine and psilocybin) to address treatment-resistant depression [5, 6]. In parallel, non-pharmacological treatments, including neuromodulation techniques, have gained prominence as strategies to improve outcomes in difficult-to-treat depression [7]. Besides antidepressant medication, light therapy, sleep-deprivation, electroconvulsive therapy and movement therapy are possible non-pharmacological treatments with a level A or B recommendation. Level A refers to at least one high-quality meta-analysis with overall consistency of results,Level B refers to at least one high-quality systematic review with consistency of results according to the Association of the Scientific Medical Societies (AWMF following the Oxford Centre for Evidence Based Medicine (OCEBM. Among neuromodulation approaches, electroconvulsive therapy (ECT remains the most efficacious for severe or refractory cases [8] (Level A evidence), but its cognitive side effects and limited patient acceptability drive the search for other options. Repetitive transcranial magnetic stimulation (rTMS) of the prefrontal cortex has emerged as a less invasive alternative that can modulate brain activity [9]. The 2022 German National Care Guideline (Bundesärztekammer (BÄK), Kassenärztliche Bundesvereinigung (KBV), Arbeitsgemeinschaft der Wissenschaftlichen Medizinischen Fachgesellschaften (AWMF). Nationale Versorgungsleitlinie Unipolare Depression—Langfassung, Version 3.2. 2022. 10.6101/AZQ/000505) provides a level B recommendation for the use of rTMS in treatment-resistant depression. In fact, high-frequency rTMS of left dorsolateral prefrontal cortex (DLPFC) shows definite antidepressant effects at the group level (level A recommendation) but its clinical efficacy is limited due to high inter-individual variability with response rates around 30% in real-world settings [9–12]. More recent consensus reviews with large-scale real-world studies report response rates up to over 50–60% depending on patient characteristics and treatment parameters [13] Recognizing this heterogeneity, experts emphasize that rTMS protocols should be tailored whenever possible and it is strongly advised that the choice of stimulation site and type is made at a specialized center, which is a level A recommendation [11, 14]. This points out the lack of a consensus regarding the use of different TMS protocols at the individual level and underscores the importance of addressing the existing heterogeneity by the advancement of personalized TMS treatment paradigms. One such FDA-cleared innovation is intermittent theta-burst stimulation (iTBS), a patterned form of rTMS delivered in only ~ 3 min per session. A landmark non-inferiority trial demonstrated, that iTBS to left DLPFC achieves antidepressant outcomes equivalent to traditional 10 Hz rTMS (≈37-min sessions) [15]. Thus, iTBS offers a time-efficient alternative with similar clinical benefit. In practice, iTBS is increasingly adopted as a standard protocol for MDD, enabling higher patient throughput and the possibility of accelerated treatment courses (e.g., through multiple-session-daily designs) in recent trials and reviews [13, 16]. Despite these advances, the field continues to seek ways to improve remission rates by optimizing how and e.g. *when* brain stimulation is delivered for each patient [17, 18].

Critically, MDD is not merely a psychological or chemical imbalance, but is associated with altered brain network activity and dysfunctional neural oscillations [19, 20]. Electroencephalography (EEG) provides an excellent basis for the development of biomarkers due to its high temporal resolution [21], which makes it an ideal tool for detecting these subtle but significant changes in real-time. For example, increased theta-frequency oscillations in frontal and limbic circuits have been linked to depressive pathology [18]. In the international Study to Predict Optimized Treatment in Depression (iSPOT-D), a large international multi-center randomized prospective practical trial on predicting pharmacological treatment outcome, patients with MDD had elevated theta activity in both frontal cortex and anterior cingulate cortex, which was associated with non-response to pharmacological treatment, whereas low theta activity was associated with treatment response, suggesting that excess frontal theta is a marker of an active, treatment-resistant depression network [22]. In contrast, patients with MDD did not differ from controls on frontal alpha asymmetry in this study [23]. This and other studies support frontal midline theta activity as a potential biomarker of MDD severity and prognosis [24–26]. The presence of abnormal frontal alpha asymmetry is also discussed as a biomarker indicative for MDD [27]. Overall, EEG-based measures offer a window into the dysfunctional brain circuitry of MDD and are being actively explored as objective biomarkers to guide and evaluate treatments.

The here proposed trial links these findings to the core hypothesis: that targeting and modulating these pathological brain oscillations in real-time may represent a more effective and personalized treatment strategy. A promising strategy to personalize rTMS is to time stimulation pulses in accordance with the patient’s intrinsic EEG rhythms [28, 29]. The physiological rationale is that brain networks cycle through fluctuating excitability states, and stimulating at specific phases of an ongoing oscillation can maximize neural impact [17, 28]. In the motor cortex, brain oscillation-synchronized high-frequency bursts of TMS, i.e., TMS triggered to the trough of the instantaneous sensorimotor µ-oscillation as detected by real-time EEG analysis, has been shown to consistently increase motor cortical excitability, similar to long-term potentiation, while targeting the positive peak of the µ-oscillation or random phase stimulation did not show this effect [30]. These findings suggest that the full therapeutic potential of rTMS to modulate dysfunctional brain networks can be more effectively exploited by personalizing rTMS therapy to individual brain states, such as brain oscillations. Exploiting this newly developed EEG-TMS technique, we have conducted a single-session proof-of-principle study to investigate the feasibility, safety and immediate neurophysiological effects of EEG-triggered brain alpha-oscillation-synchronized TMS of left DLPFC in 17 patients with unipolar antidepressant-resistant MDD [31]. EEG-triggered brain oscillation-synchronized TMS of left DLPFC was feasible in the majority of patients (15/17, ~ 88% success rate in achieving real-time EEG triggering) and reduced resting-state alpha power in the left DLPFC (the stimulation site) compared to control conditions. Side effects reported were limited to mild discomfort at the site of stimulation. As this was a single-session proof-of-principle trial, no clinical outcome measures were obtained. These findings demonstrated that EEG-synchronized rTMS of left DLPFC can be applied safely and has the potential to interact with and modulate dysfunctional brain oscillations in patients with MDD.

Encouraged by these pilot results, research teams have begun evaluating EEG-synchronized rTMS in clinical trials. Notably, a recent double-blind randomized controlled trial examined alpha-phase-synchronized prefrontal rTMS in adults with treatment-resistant depression over a full 6-week, 30-session course [32]. In this trial 28 treated patients were randomized to receive left prefrontal rTMS either with the first pulse of each train locked to the individual’s preferred prefrontal alpha phase (“SYNC” group), or with that pulse delivered at arbitrary phases (“UNSYNC” control), with all other stimulation parameters held constant. Results showed that the SYNC condition was feasible over the prolonged treatment course and induced progressive EEG entrainment of the alpha rhythm: only the SYNC group demonstrated increased phase consistency across sessions. Furthermore, within the SYNC group, patients who exhibited stronger EEG entrainment (more consistent phase alignment session-to-session) tended to show greater clinical improvement in depression ratings. This suggests a dose–response relationship between the degree of brain rhythm modulation and antidepressant effect. However, the trial’s primary outcome found no significant difference in overall treatment response rates between the SYNC and UNSYNC groups. Both groups improved from baseline, as expected with standard rTMS, but EEG-synchronized TMS did not outperform the control condition in remission rates. The authors noted, that suboptimal timing precision in some patients and the relatively small sample might have limited the observable advantage. Nonetheless, this first RCT in the field demonstrated the feasibility of brain state-dependent TMS in a clinical setting and provided clues, that enhancing EEG entrainment could yield better outcomes. Overall, the trial reinforces the concept that EEG-synchronized rTMS can meaningfully influence brain activity in MDD, while also highlighting the need to refine synchronization.

Building upon these findings we designed the present study to rigorously test a refined EEG-synchronized rTMS intervention in MDD, guided by the converging evidence that frontal-midline theta oscillations are critically implicated in depression severity and treatment resistance. In particular, our approach focuses on the theta frequency band in the dorsomedial prefrontal cortex (DMPFC) based on accumulating evidence that elevated theta activity in medial prefrontal and anterior cingulate regions has been corelated with depression severity and resistance to conventional treatments [33–35]. Current evidence from our lab further suggests that theta activity in the left dorsomedial prefrontal cortex (DMPFC) has a higher signal-to-noise ratio than alpha activity in left DLPFC, making it a prime biomarker for targeting by EEG-triggered TMS [36]. In practical terms, the DMPFC theta rhythm may be a more robust and reliable biomarker for triggering TMS, making it an attractive target for a phase-locked stimulation paradigm. We therefore developed the BOSSFRONT2 trial to evaluate real-time EEG-triggered theta-burst stimulation of the left DMPFC as a novel therapy for treatment resistant depression. The stimulation in the experimental arm is synchronized to each patient’s endogenous frontal theta oscillation (detected via scalp EEG over DMPFC in real time), so that TMS bursts are delivered at a consistent phase of the theta rhythm. The control condition in our study is the current standard protocol: iTBS applied to the left DLPFC (the same location and protocol used in many guidelines and clinical centers). By comparing theta-synchronized DMPFC-rTMS (hereafter referred to as DMPFC-EEG-TBS) with standard open-loop iTBS (DLPFC-iTBS), our randomized double-blind trial will test whether personalized, theta oscillation-synchronized neuromodulation can achieve superior antidepressant effects. We hypothesize that engaging the pathologic theta network in a timing-specific manner will at least match, if not outperform the efficacy of conventional iTBS, while maintaining a favorable safety profile.

This pilot trial is designed as an exploratory superiority study, aiming to detect whether DMPFC-EEG-TBS yields a clinically meaningful improvement compared to standard DLPFC-iTBS. Given the pilot nature and limited sample size, the study is not powered for confirmatory testing but is intended to generate effect-size estimates necessary for planning a subsequent definitive RCT to establish the benefits of personalized, brain state-dependent TMS in MDD.

### Objectives

To study the therapeutic efficacy of four weeks (20 sessions, 5 sessions per week) of real-time EEG- synchronized rTMS of the left DMPFC compared to standard FDA-approved iTBS of the left DLPFC to reduce depressive symptoms in patients with antidepressant-resistant MDD in a controlled randomized double-blind clinical pilot trial.

### Trial design

The study is a single-site randomized controlled double-blind parallel-group clinical trial.The framework is comparative and exploratory, investigating potential superiority of the EEG-synchronized intervention over standard iTBS based on clinical outcomes.

**Fig. 1:**
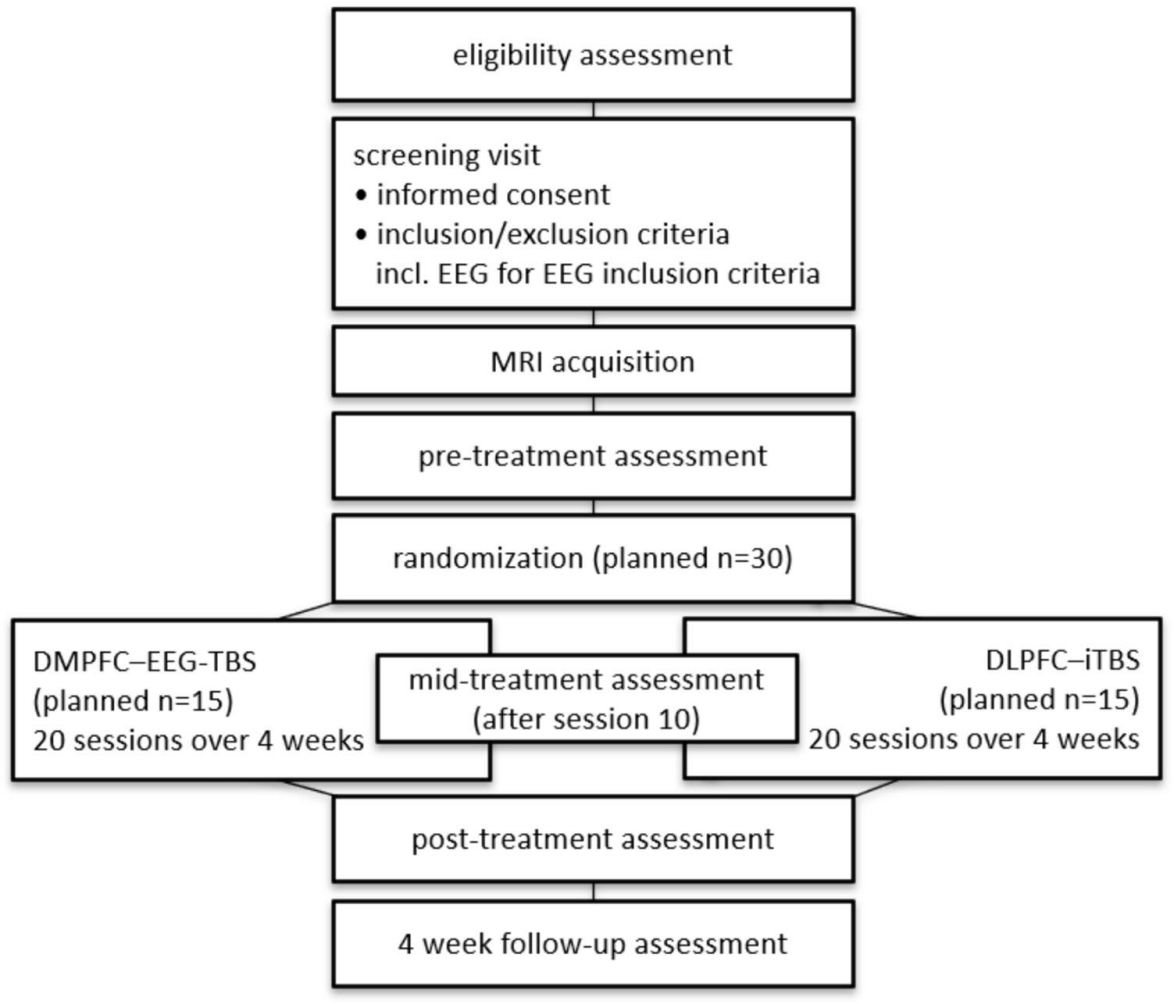
Flowchart of participant progress through the BOSSFRONT2 trial

The overall flow of participants through the trial is illustrated in Fig. 1:

## Methods: participants, interventions, outcomes

### Study setting

The study will be conducted at the Dept. of Psychiatry and Psychotherapy at the University Hospital of Tübingen. All treatments will be performed in a suitable experimental location with a qualified medical doctor available on site. The study is planned to span a total duration of 24 months.

### Eligibility criteria

Subject inclusion criteria.

- Subjects are between 18 to 65 years old
- Subjects meet DSM-5 criteria for current major depressive disorder (MDD), confirmed with the Structured Clinical Interview for DSM-5.
- Subjects score 20 points or more on the Montgomery–Åsberg Depression Rating Scale (MADRS).
- Subjects must have had at least one non-response in accordance with the Antidepressant Treatment History Form (ATHF), meaning a failure to achieve remission in a previous pharmacological antidepressant treatment trial of sufficient dosage and duration, meaning a dose considered to be effective (e.g., superior to placebo in controlled clinical trials) and a duration needed to be sufficient to produce a robust therapeutic effect (e.g., 4–6 weeks); this treatment failure can apply for the current or any prior depressive episode. Demonstration of medication resistance for the current episode is not required, if resistance occurred during any prior depressive episode.
- Subjects must exhibit a sufficiently detectable frontal-midline theta rhythm during the EEG screening procedure. This is assessed with a 32-channel resting-state EEG (5 min, eyes open) conducted at the screening visit. Theta power spectra are computed and visually inspected for the presence of a clear peak in the 4–8 Hz range over midline frontal electrodes. Only patients with a discernible theta peak are eligible, as this is required to ensure feasibility of the real-time algorithm.
- Subject is in otherwise good physical and mental health. Subject understands the study procedures and agrees to participate in the study by giving written informed consent.
- Subject is willing to comply with the study restrictions (cf. “paragraph relevant concomitant care …”)
- If antidepressant medication is being taken, it has to be taken for at least 2 weeks before inclusion in the study and the dose or active substance must not have been changed. It is necessary that no change in medication will be made until the end of the study (last visit takes place 4 weeks after the last therapy session). If a change in medication is necessary, further study participation is not possible.

Subject exclusion criteria:

- Subject is under the age of legal consent.
- Subject has a diagnosis of bipolar disorder.
- Subject suffers from current symptoms of psychosis.
- A current major depressive episode longer than 5 years.
- Subject has a history of substance abuse or dependence within the past 2 years.
- Subject has a diagnosis of antisocial or borderline personality disorder.
- Subject has active suicidal ideation with plan and/or intent.
- Subject has a history of seizure disorder.
- Subject has a history of severe head injury with loss of consciousness.
- Subject had a prior brain surgery, or any other major psychiatric or medical comorbidity.
- Subjects with intake of pro-convulsive medication, e.g. imipramine, amitriptyline, doxepin, nortriptyline, maprotiline, chlorpromazine, clozapine, foscarnet, ganciclovir, ritonavir, amphetamines, cocaine, MDMA (ecstasy), phencyclidine (PCP, angel’s dust), ketamine, gamma-hydroxybutyrate (GHB), alcohol, theophylline, in accord with the present consensus guideline on safety, ethical considerations, and application of TMS in clinical practice and research [37, 38].
- Daily intake of benzodiazepines in equivalent doses of lorazepam > 1 mg/d.
- Subject has a cardiac pacemaker, implanted medication pump, intracardiac line, or acute, unstable cardiac disease.
- Subject has an intracranial implant (e.g., aneurysm clips, shunts, stimulators, cochlear implants, or electrodes) or any other metal object within or near the head (excluding the mouth) that cannot be safely removed.
- Subject has participated in another study within 2 weeks prior to the first study visit.
- Subject has contraindications to MRI, or does not agree that (1) the MRI is obtained for research purposes only and will not be evaluated by a radiologist; if an abnormality is present, this may well not be noticed by the doctors, scientists and other staff involved in the study and handling the MRI data; and that (2) if any of the staff involved in the study does suspect a relevant abnormality to be present in the MRI, they will reveal this to the subject so that a further diagnostic workup can be conducted outside of the study.
- Subject is pregnant or trying to get pregnant. If someone is not sure whether she is pregnant we will test human chorionic gonadotropin in the urine.
- Planned or anticipated changes of medication within the study period. If a change in medication is necessary, further study participation is not possible.

### Who will take informed consent?

Patients will be checked for eligibility by a study physician. Eligible patients will be informed about the modalities of the clinical investigation in accordance with the provided patient informed consent (IC). The patient is to be informed both in writing and verbally by the investigator before any study‐specific procedure is performed. The patient will be given sufficient time (i.e., > 24 h) to decide whether to participate in this study and to ask questions concerning this trial. The patient must give consent in writing. The patient and informing physician must each personally date and sign the informed consent form with an integrated declaration on data privacy protection.

### Additional consent provisions for collection and use of participant data and biological specimens

Not applicable.

## Interventions

### Intervention description

**Fig. 2:**
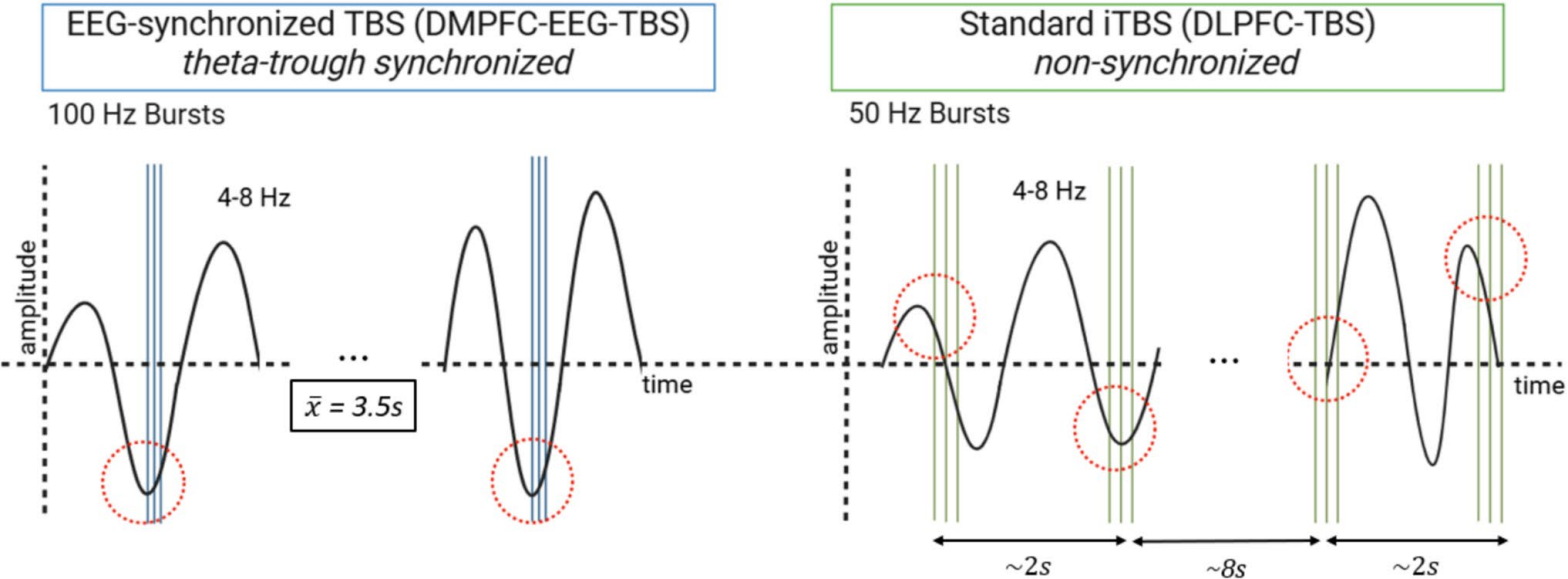
Schematic comparison of the experimental EEG-synchronized theta-burst stimulation (DMPFC-EEG-TBS) and the control condition with standard iTBS (DLPFC-iTBS)

Subjects will be randomized to one of two arms of daily rTMS interventions for four weeks (5 sessions per week on working days, 20 sessions in total). In the experimental EEG-synchronized condition, TMS will be triggered at the trough of the theta oscillation extracted from left DMPFC. The control condition will consist of rTMS therapy applied to the left DLPFC following an FDA approved iTBS protocol [15] (s. Figure 2):

1. Experimental arm (DMPFC-EEG-TBS):

Triple pulses at 100 Hz are delivered in bursts phase-locked to the trough of the individual theta oscillation (4–8 Hz) recorded over the left dorsomedial prefrontal cortex (DMPFC) (s. figure 2). Although rising or falling phases of theta oscillations may transiently exhibit higher instantaneous excitability, previous work has demonstrated that stimulation precisely at the trough yields the most reliable and long-lasting plasticity-like effects [30, 31, 39]. By adopting 100 Hz we rely on evidence, that higher intraburst frequencies strengthen plasticity-like effects within established safety margins [40, 41]. Extending this rationale, frequency-dependence has been shown with state-dependent bursting in motor cortex, where 100–200 Hz bursts at the trough of the ongoing μ-rhythm reliably increased cortical excitability, whereas 60 Hz did not [39]. In our protocol, bursts are phase-locked to the trough of the endogenous frontal theta oscillation over left DMPFC to exploit a receptive excitability state for plasticity induction [30]. Bursts are triggered on roughly every 15-20th trough, yielding a mean inter-burst interval of approximately 3.5 s, consistent with our prior prefrontal real-time work reporting 3.49 s (SD ± 2.01) {Gordon, 2022 #81}. A minimum inter-burst interval of 1.0 s is enforced to ensure artifactfree phase estimation following each burst. Under these constraints the session delivers 600 pulses and lasts about 11 min 38 s, as shown in Figure 2.

2) Control arm (DLPFC-iTBS):

Standard intermittent theta-burst stimulation (iTBS) is applied to the left dorsolateral prefrontal cortex (DLPFC) according to FDA-approved parameters (50 Hz triplets repeated at 5 Hz, 2 s trains followed by 8 s pauses) until 600 pulses are delivered, resulting in a total duration of about 3 min 10 s.

In the experimental arm, bursts of three pulses at 100 Hz are phase-locked to the trough of the ongoing theta oscillation (4–8 Hz), ensuring synchronization with intrinsic brain rhythms. Circles highlight stimulation timing relative to the oscillatory phase. Of note, in the experimental condition, stimulation is not delivered on every detected theta trough but occurs with a mean inter-burst interval of approximately 3.5 s, corresponding to every 15th–20th theta cycle. In the control arm, bursts of three pulses at 50 Hz are delivered in a fixed 5 Hz pattern independent of the EEG signal (open-loop). Hereby each session in the control condition consists of 600 pulses applied in 2 s “on” / 8 s “off” cycles.

In both interventions, 600 TMS pulses will be delivered per session corresponding to 12.000 TMS pulses during the entire 4 weeks of TMS therapy. Stimulation intensity will be set to 120% resting motor threshold (RMT) or the highest tolerated intensity if this is below 120% RMT. If required, a ramp-up period will be used for a maximum of the first three sessions to accommodate patients that don’t immediately tolerate the desired stimulation intensity. Stereoscopic neuronavigation (Localite GmbH, Bonn, Germany) based on individual anatomical MRI data will be used to position the coil, where DMPFC and DLPFC target locations will be pre-determined based on the individual MRI data and identifying the respective target gyrus using standard anatomical methods [42].

To ensure blinding of the patients, all setup components are implemented identically in both conditions, that is, a surface EEG is recorded through a 5-channel equipment during the TMS intervention in both the experimental and in the standard condition. But only in the experimental condition is the EEG used to trigger the TMS pulses; in the control condition, the EEG is recorded but not considered for triggering the TMS pulse. To ensure double-blinding, the rater is blinded as well, as the rater must not be present during the intervention procedure and does not have access to non-rater-related documentation.

### Measurement procedures

- MRI

The MRI images will be acquired at a Siemens 3 Tesla MRI scanner in the MRI Research Center of Tübingen (Department Biomedical Magnetic Resonance, Prof. Dr. phil. nat. Dipl.-Phys. Klaus Scheffler, Hoppe-Seyler-Str. 3, 72076 Tübingen). Patients are placed in the scanner with earplugs and an emergency ball. Visual and verbal contact to the patients is maintained from the control room. No drugs or contrast agents are used during MRI examinations.

Study participants are evaluated by a medical doctor for MRI contraindications and need to give written informed consent before the scan. Subjects are required to give written consent that if the scan reveals a gross anatomical abnormality, they will be informed and need to take further diagnostic work-up outside of the study. Acquired scans are performed for scientific reasons with parameters optimized according to the requirement of the stimulation and thus do not provide sufficient diagnostic value in case of structural abnormalities.

- rTMS

Study participants will be seated on a comfortable reclining chair with both arms relaxed. A high-frequency capable TMS stimulator (MagPro XP, MagVenture, Denmark) with a figure-of-eight coil (Cool-B65, inner coil winding diameter 35 mm) and a TMS-compatible EEG setup (ActiCHamp, BrainProducts, Gilching, Germany) will be used. For DMPFC-EEG-TBS, TMS pulses will be triggered from a custom real-time digital biosignal processing system based on the ongoing oscillatory theta brain activity in the left DMPFC as recorded by surface EEG.

- Stereoscopic Neuronavigation

Stereoscopic neuronavigation (Localite GmbH, Bonn, Germany) will be used to track the position of the coil relative to the head and to determine the appropriate stimulation sites (DMPFC and DLPFC, respectively).

- EEG

EEG recordings will be carried out using a TMS-compatible, optically isolated amplifier (ActiCHamp, BrainProducts, Gilching, Germany).

For the theta screening and the pre- and post-assessment surface EEG series will be recorded for 5 min with eyes open using a TMS-compatible 32-channel gel filled sintered ring electrode EEG cap (ActiCap, BrainProducts, Gilching, Germany) using the same optically isolated amplifier as described above. Electrode impedances are kept below 10 kΩ, the sampling rate is set to 1,000 Hz with 24-bit resolution, and FCz serves as the reference electrode. The primary purpose of the screening EEG procedure is to evaluate the presence of a detectable theta rhythm in the 4–8 Hz frequency range over midline frontal electrodes. Spectral power is computed in real time, and a graphical representation of the frequency spectrum is generated for visual inspection. A clearly identifiable theta peak is required for study inclusion, as the EEG-synchronized stimulation depends on reilable phase detection in this frequency band. This screening step ensures the technical feasibility of the intervention and serves as an additional eligibility criterion. In addition to the screening EEG, resting-state EEG is also recorded immediately before the start of treatment (pre-assessment) and after the final stimulation session (post-assessment). The purpose of these recordings is to evaluate changes in spectral power and functional connectivity across treatment, with a focus on the theta frequency band. Exploratory analyses will also investigate alterations in other frequency ranges and potential shifts in frontal network dynamics.

For the stimulation sessions, a five-channel setup is employed with an AFF1h-centered Hjorth montage (AFF1h, AFF2h, AFp1, FFC1h, AFF5h) to capture frontal theta oscillations over the left dorsomedial prefrontal cortex (DMPFC) {Gordon, 2021 #12}. EEG is recorded with the identical montage and equipment in both conditions as described above, but is not used to control the timing of stimulation in the control condition, thereby maintaining blinding while also enabling exploratory offline analyses.

- Real-time EEG algorithm

The real-time pipeline consists of a band-pass filter in the theta range (4–8 Hz), a 50 Hz notch filter to remove line noise, and continuous monitoring for artifacts. Theta oscillations are extracted from the AFF1h channel, and instantaneous phase is computed using a Hilbert transform with latency compensation. TMS bursts are triggered at the trough of the theta cycle, with prediction to compensate the fixed delay of 15–20 ms arising from amplifier buffering, signal processing, and stimulator triggering; the predictor advances the command by ~ 30–45° at 6 Hz [30]. To allow undisturbed phase estimation, a hard refractory period of 1.0 s is imposed after each burst. Thereafter, the next eligible trough is used, selecting every 15-20^th^ trough to obtain a mean interburst interval ~ 3.5 s. Session level phase-locking metrics are computed online to verify timing accuracy (see also Fig. 2). This calibration procedure follows established practice in phase-specific TMS research [30] and recent delay analyses highlighting uncompensated transport latency as a critical determinant of phase-locking accuracy [43]

- EMG/MEP

Surface electromyography (EMG) will be obtained through an optically isolated battery powered biosignal amplifier (ActiCHamp, BrainProducts, Gilching, Germany) using bipolar electrodes from right hand muscles (first dorsal interosseous and abductor pollicis brevis muscles). This enables determining the RMT, which is needed to individualize stimulation intensities during the rTMS protocols. RMT is defined as the minimum stimulus intensity needed to evoke motor evoked potentials (MEP) of > 50 µV peak-to-peak amplitude in the target muscle in at least 5 out of 10 consecutive trials using a figure-of-eight TMS coil oriented 45° away from the midline over the hand representation of left primary motor cortex [44]. Patients will be requested to relax and stay awake during the experiments.

- Clinical Assessments

Clinical assessments regarding the severity of the depression before and after the TMS treatment will be performed by a blinded rater using the MADRS and HDRS-17 scales.

- Self-Report

Subjects will be asked to complete the Beck-Depressions-Inventory-II (BDI-II) (this will take 5 min) and the Inventory of depressive symptoms-30 (IDS-30) (this will take 10 min) at baseline (pre-assessment), after the 10th session (intermediate assessment), after the last session (post-assessment) and at the follow-up visit four weeks after the last intervention (follow-up assessment). Following each intervention session, a structured self-report will be documented, to assess whether any side-effects occurred from the stimulation. Side effects will be documented on a separate adverse event sheet.

### Criteria for discontinuing or modifying allocated interventions

If the stimulation is not tolerated by the patient, the stimulus intensity will be first adjusted to the highest tolerated intensity and then gradually adjusted to the required intensity over max. 3 sessions. If the patient cannot become acquainted with the treatment conditions (e.g., stimulus intensity) within three sessions, additional participation is not feasible. Withdrawal from the study is also required if the subject misses more than 4 planned treatment sessions. In the case of up to 4 missed planned treatment sessions, the missed sessions will be offered at the end of the intervention period, so that each enrolled patient will receive 20 sessions. However, we strive to keep the number of treatment cancellations and postponements to a minimum. Patients who withdraw from the study will be included in the primary statistical analysis (cf. section “Statistical Methods”).

### Strategies to improve adherence to interventions

Adherence to the intervention is considered to be high as patients decide to participate in this brain stimulation study on their own initiative. TMS treatment is non-invasive, painless and is generally considered to be well tolerated. Participation adherence is expected to be also increased by the ramp-up method with a familiarization phase to the stimulation intensity (if required) and the possibility of 4 missed planned treatment appointments.

### Relevant concomitant care permitted or prohibited during the trial

No specific antidepressant medication is mandatory. However, if present, the antidepressant therapy must be kept constant, which means that medication at or above the “minimum oral dose” (MOD) specified in the Antidepressant Treatment History Form: Short-Form (ATHF-SF) [45] has to remain unchanged 4 weeks before and 6 weeks during study treatment. Changes below the MOD are considered as uncritical. Changes after the end of treatment are registered in the follow-up sessions. Critical changes during the treatment period lead to exclusion from the per protocol analysis.

Patients are advised not to undergo any concomitant therapies if possible. In the case of ongoing therapies (e.g., psychotherapy), patients are asked to keep these constant in intensity and frequency throughout the study period.

### Provisions for post-trial care

Ancillary treatment is not planned during the intervention period due to confounding influence. In case of severe mental health conditions (e.g., suicidal ideation), requiring immediate standard of care treatment (inpatient, psychotherapeutic), the patient will be transferred to treatment by the established infrastructure. In the same manner, post-trial care is provided in case of severe disease aggravation by the end of trial participation.

If a participant withdraws prematurely from the study, their reasons, circumstances, and final status will be documented where possible. If the patient does not withdraw the consent for further follow‐up, he/she will be followed‐up as planned. If any harm arises from participation, such as an epileptic seizure or headache, standard treatment for the symptoms will be provided at the hospital immediately.

No post-treatment taper phase is included within the present protocol, since the trial was designed to closely mirror the FDA-approved iTBS standard and the addition of taper sessions was not feasible within the capacity of a pilot study. However, outside the study, participants have the opportunity to receive further treatment in our Day Clinic for Brain Stimulation, where standard iTBS is routinely available as part of clinical care. This option does not constitute a study-related provision but reflects the regular treatment pathways available to all patients. This ensures that participants who require continuation therapy can receive it under standard clinical conditions.. Future confirmatory trials will address this issue by incorporating taper phases or maintenance protocols, thereby providing a more complete picture of how oscillation-synchronized TBS can be implemented in clinical practice.

## Outcomes

### Primary endpoint

The primary outcome measure of the study is the change in the Montgomery-Åsberg Depression Rating Scale (MADRS) after the last interventional session (4 weeks after baseline) compared to at the baseline measurement before the first treatment session. The Montgomery-Åsberg Depression Rating Scale is a questionnaire for external assessment of the severity of a depressive syndrome. The questionnaire consists of 10 questions that are rated on a 7-point scale from 0 to 6. The total score ranges from 0 (best outcome) to 60 (worst outcome) points. The questionnaire is considered gold standard in the assessment of depressive symptoms [46].

### Secondary endpoints

1. Change in MADRS 4 weeks after the last treatment session vs. measurement before the first treatment session.
2. Response rate after the last treatment versus baseline measurement before the first treatment session. A response is defined as a 50% reduction in the MADRS score from baseline [47].
3. Remission rate after the last treatment versus baseline. measurement before the first treatment session. Remission is defined by a MADRS score below 8 points [47].
4. Change in HDRS-17 (Hamilton Depression Rating Scale-17) after the last treatment session vs. measurement before the first treatment session. The HDRS-17 rates the severity of depressive symptoms in patients based on 17 structured interview questions performed by a clinican. A score of 0–7 is considered to be normal while a score of 20 or higher indicates at least moderate severity [48].
5. Change in BDI-II (Beck Depression Inventory-II) after the last treatment session vs. measurement before the first treatment session. The BDI-II is a 21-question multiple-choice self-report inventory and represents the most widely used psychometric test for assessing the severity of depression in patients. More than 29 points reflect high severity of depressive symptoms, whereas a score below 13 points reflects mild depressive symptoms [49] [50].
6. Change in IDS-30 (Inventory of depressive symptoms-30) after the last treatment session vs. measurement before the first treatment session. The IDS-30 is considered equivalent to or superior to the standard Hamilton Depression Scale (HDRS) and Beck Depression Inventory (BDI) tests. The scores ranges from 0 to 84 points. Patients who score from 39 upwards are considered severely depressed [51].

### Exploratory end points

Exploratory outcome measures will be changes in resting-state EEG immediately after and 4 weeks after the last treatment session vs. a baseline measurement before the first treatment session. In particular, the power spectrum density function derived from the 5-channel AFF1h-centered Hjorth montage and functional connectivity from the stimulated AFF1h electrode to all other EEG electrodes, using the weighted phase lag index in the theta frequency band, will be explored.

### Participant timeline

The TMS intervention duration for each subject is 4 weeks (20 sessions, 5 sessions per week). The maximum study duration including screening, MRI scan, TMS intervention and a 4-weeks follow-up measurement for each subject will be 3 months. The time course of events can be seen in Table 1.

**Table 1:**
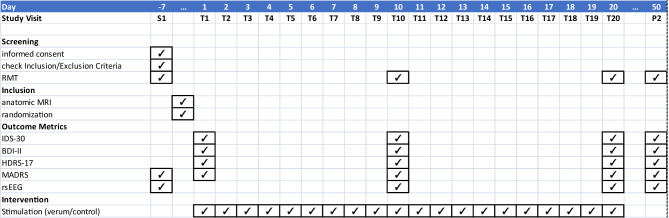
Study Visits with clinical assessment timeline and diagnostic scores per treatment phase

There will be three types of study visits which will be described in detail below:

- Screening Visit
- Assessment Visit
- Therapy Visit

#### Screening visit

After having obtained written informed consent the screening visit consists of checking for inclusion/exclusion criteria incl. MRI safety checklists, physical and psychiatric examination and scheduling the MRI scan.

The screening visit also includes a resting-state EEG measurement (32 channels, 5 min, eyes open). This EEG is analyzed for spectral power in the theta range (4–8 Hz). A clear theta peak must be present to qualify for study participation, as this feature is necessary for the functioning of the real-time EEG-triggered stimulation algorithm. The spectral analysis is presented graphically, and eligibility based on this criterion is confirmed by senior investigators.

A second brief screening visit will be scheduled within 3 days prior to the first scheduled therapy visit to ensure that the patient still fulfills the inclusion criteria. An MRI measurement will be scheduled between the two screening visits. The stimulation sites will be marked. Resting Motor Threshold determination and randomization will also be performed in this visit.

#### Assessment visits

Each patient will receive four evaluations: a pre-evaluation (T1; prior to the first intervention), an intermediate evaluation (T10; after the 10th intervention), a post-evaluation (T20; after the final intervention), and a follow-up evaluation (P2) conducted four weeks after the last intervention.

These assessments will include clinical evaluations (MADRS, HDRS-17), self-report questionnaires (BDI-II, IDS-30), a 32-channel EEG, and TMS measures of motor cortical excitability (RMT). The randomization is performed between the pre-evaluation and the first treatment session.

#### Therapy visit

A 5-channel EEG cap with an AFF1h-centered Hjorth montage (AFF1h AFp1, AFF2h, FFC1h, AFF5h) with active electrodes using ActiCHamp (BrainProducts GmbH, Gilching, Germany) will be prepared, followed by registration of the MRI-based TMS-navigator. In both conditions MRI-navigated TMS with 600 pulses per session will be applied to the prefrontal cortex. The intervention protocol (EEG-synchronized rTMS over left DMPFC or standard iTBS over left DLPFC) depends on the randomization. The patients will not be informed about their assignment to the experimental or control protocol.

At the start of each study visit, patients will be asked about changes in medication. The patient is asked about the occurrence of any adverse events at the end of each therapy visit and again at each subsequent study visit to account for adverse events that may have occurred between sessions. In the case of adverse events, any event will be documented in the case report form, a study physician will be consulted and feedback is given to the principal investigator and the sponsor. The principal investigator decides on the severity of the adverse event and further study participation in consultation with the patient. In case of changes in the medication, the patient must be excluded from further participation.

### Sample size

The primary outcome measure is defined as the percentage reduction of the depressive symptoms (MADRS) between baseline and after the last treatment session for each patient. The following assumptions are made on the basis of available data: an assumed improvement in the MADRS of 48.0% (SD of 17.9) for standard TMS therapy [52]. Justification for a subsequent confirmatory trial should be shown if the improvement in the MADRS will be 20% or even more (SD of 17.9). This means that the reduction in the MADRS will be 68% in the intervention group. Under these conditions, a two-sided independent t-test applied to a sample size of n = 13, 6 in each group will have 80% power for refusal of the null hypotheses that the efficacy of both therapies is not different (significance level α = 0.05). Assuming a drop-out rate of 20%, a total of n = 34 patients should be randomized to reach at least a number of 28 assessable patients. Because the present study is conceived as an exploratory pilot, we retain the originally planned sample size of N = 28 to obtain preliminary effect-size estimates and feasibility data necessary for planning a subsequent confirmatory RCT. The sample size determination was established using SAS 9.4 Proc Power (SAS Institute Inc., Cary, NC, USA).

### Participants and recruitment

Thirty patients with MDD as diagnosed by the Diagnostic and Statistical Manual of Mental Disorders (DSM-5) in the age range of 18–65 years will be included. Potential candidates will be addressed by contacting local patient advocacy/support groups, local psychiatrists/general practitioners, as well as information via clinic website, social media, newsletters, local newspapers, or advertisement in public transportation.

Both men and women are eligible. Gender and age distribution will be documented and reported in the trial results. While the study is not powered to detect gender-specific effects, exploratory analyses will be undertaken to investigate potential trends in treatment response by gender. These analyses may provide preliminary data for subsequent pooled or confirmatory trials.

## Methods: assignment of interventions

### Allocation, sequence generation and implementation

Participants will be randomly allocated to either the control or experimental group in a 1:1 ratio based on a computer-generated randomization list provided by The Institute of Clinical Epidemiology and Applied Biostatistics at the University of Tübingen (IKEaB). The randomization will be provided in envelopes that indicate the assigned stimulation protocol. The operator is responsible for implementing the assigned treatment while ensuring the patient remains blinded.

### Blinding

Assessments of the clinical endpoints will be conducted by an assessor blind to treatment allocation. Participants and assessor will be blinded to the allocation. The therapy provider cannot be blinded to allocation due to the nature of the intervention, but is encouraged not to disclose the allocation status to the patient or the assessor.

Procedure for unblinding if needed:

To maintain the overall quality and legitimacy of the clinical trial, unblinding should occur only in exceptional circumstances when knowledge of the actual treatment is absolutely essential for further management of the patient. Investigators are encouraged to discuss with the principal investigators if unblinding is necessary. If unblinding is necessary, the investigator should use the system for emergency unblinding. However, no scenario can yet be identified in which unblinding would be medically necessary. Given that both therapy arms include effective TMS and that the therapies do not differ medically in terms of their risk profile, in this study unblinding would not have a therapeutic consequence.

The Investigator is encouraged to maintain the blinding as far as possible. The actual allocation must not be disclosed to the patient and/or other study personnel including other site personnel, monitors, corporate sponsors or project office staff; nor is there any written or verbal disclosure of the code in any of the corresponding patient documents. The investigator reports all unblinding (with reason) to the data management department at the IKEAB (Institute for Clinical Epidemiology and applied Biostatistics, University Tübingen).

## Data collection, management and analyses

### Plans for assessment and collection of outcomes and data management

The informed consent process is documented and all the original signed documents will be part of the investigator’s site file and retained with it. One copy including the insurance policy of the trial will be handed to the patient. All procedures administered to the subjects on entry to the trial or at any time during the trial will be documented paper-based in a pseudonymous manner. At the end of each visit, the data will be entered into a paper-based CRF. The CRFs have been validated and approved by the IKEaB.

The investigator’s site file will be stored in the TMS study center of the University of Tübingen in accordance with the regulatory requirements of General Data Protection Regulation (GDPR) directives. Only authorized staff will have access to the data. All source data will be photocopied, pseudonymized and digitally stored on the secured storage area network (SAN) of the University Hospital Tübingen. Subjects’ files will be saved using codes in order to protect their privacy. Volume cloning ensures instantaneous data backup. Publication of data, e.g. in the form of a scientific oral presentation or publication will only contain anonymized data. MRI data will also only be saved in a pseudonymized way. Data will be entered in a database (www.koordobas.de) and checked for errors, consistency and completeness before submitting to the statistical analyses. All data will be stored for a minimum of 10 years to enable data reanalysis and sustained availability.

### Confidentiality

Data acquisition and storage is performed in accordance with the regulatory requirements of General Data Protection Regulation (GDPR) directives. CRFs or other data that might be photocopied for verification by authorized persons will be pseudonymized, i.e., they will not contain the name of the participants but only their unique identification code. The identification code consists of an abbreviation of the study acronym (BF2) plus the number of the participant. Published data (e.g., in form of scientific oral presentations or publications) will only contain anonymized data. Participants’ files will be saved using codes in order to protect their privacy.

### Plans for collection, laboratory evaluation and storage of biological specimens for genetic or molecular analysis in this trial/future use

Not applicable, no samples collected.

## Statistical methods

### Methods and analyses

All statistical analyses will be based on the Intention-to-Treat Population (ITT). The ITT includes all randomized patients with exception of patients who withdraw their informed consent for the analysis of their data during the study. Additionally, as per protocol analysis of main results will be provided, if ITT differs from PP by more than four patients.

The primary endpoint is the relative change (%) of MADRS between baseline and the last therapy session. The primary endpoint will be compared between the two study groups. The null-hypothesis is that there is equal relative change of MADRS in both groups. The alternative hypothesis is that the experimental group has a greater relative change of MADRS. For analysis of the primary endpoint variable a baseline adjusted analysis of covariance will be performed to test the null hypothesis against the alternative hypothesis. The test of the factor “treatment” will be based on a significance level of α = 0.05 one-sided. Effect size and 95%-confidence interval for effect size will be estimated. The rejection of the null hypothesis will be interpreted as justification to plan a confirmatory RCT. Additionally, a mixed model will be calculated including all measurements of MADRS with study arm and time as factors and the time vs treatment interaction as parameter of interest. In this analysis, screening and measurement before treatment at T1 will be pooled.

All secondary outcome measures will be compared and statistically assessed for descriptive purposes and not in a confirmatory sense. The aim of these analyses is explorative, not hypothesis testing or evidence for efficacy generating, and no attempt will be made to adjust the p-values of statistical tests of the secondary endpoints for multiple testing. If adequate, secondary endpoints will be compared and statistically assessed using t-tests for two groups or analysis of covariance techniques with baseline values as covariates in case of quantitative variables. Depending on distribution, non-parametric tests may be indicated. Dichotomous data will be compared and statistically assessed using Mantel–Haenszel chi-squared tests including relative risks and 95%-confidence intervals for relative risks and by using logistic regression models.

All variables included in the CRF are mandatory. The monitoring will assure quality of the assessments. Thus, missing values are to be expected only due to refusal by patients. Missing values will be simulated using multiple imputation. The percentage number of sessions (at most 20 = 100%) will be used as a covariate, this means, in patients with zero sessions, jump to reference will be applied. Zero % change, complete case and last observation carried forward analyses will be performed as sensitivity analyses.

Safety will be assessed by frequency tabulations and line listings, and exact 95% confidence intervals if adequate.

Descriptive analyses will include absolute and percentage frequencies for categorical variables, means, medians, standard deviations, quartiles and ranges for quantitative variables and medians, quartiles and ranges for ordinal variables.

No subgroup and no interim analyses are planned. There are no stopping rules to define. Due to the missing interim analyses there are no criteria for an early stop of the clinical trial. If a patient worsens during the treatment period this could be defined as an individual reason for an early drop out of the study.

Statistical analyses will be conducted by the Institute for Clinical Epidemiology and applied Biostastistics of the University of Tübingen, once database is declared closed. In this study protocol the outline of the planned analyses are given. Before starting the final analysis, a detailed statistical analysis plan will be written and signed by the responsible statistician and the coordinating investigator. If any major deviations in the SAP from the original study protocol are necessary, the reason for that will be given in detail. A protocol amendment will be adequate for major deviations (e.g., change of primary endpoint, sample size). The final SAP will be documented in the final biometrical report. The biometrical report will be written by the responsible statistician and should include the complete statistical analysis.

## Oversight and monitoring

### Adverse event reporting and harms

Adverse events will be assessed in each study visit and documented. The reporting of AEs will be in accordance with standardized procedures of the regulatory requirements. The Common Terminology Criteria for Adverse Events (CTCAE) criteria (version 5.0) will be used to assess the severity of the AEs.

All experiments will be performed in a suitable experimental location in the Dept. of Psychiatry and Psychotherapy at the University Hospital of Tübingen with a qualified medical doctor available on site. Standard safety precautions to monitor the risk of possible induction of seizures will be followed, according to the present consensus guidelines on safety, ethical considerations, and application of TMS in clinical practice and research [37].

Magnetic Resonance Imaging (MRI) is a daily-applied diagnostic method. Severe effects of magnetic fields and radio frequency are not known, as long as the standardized MRI inclusion/exclusion criteria are obeyed. Possible side-effects are muscle twitching or irritation of the nerve while the patient is inside of the MRI-scanner. Other side-effects are headaches and tinnitus as well as overwarming and heating of metallic tattoos.

The most severe acute adverse effect for rTMS is the risk of the induction of seizures. However, in the more than 1000 papers published using rTMS between 1999–2020 twelve seizures were reported, one of which may have been a pseudo-seizure, three could have been syncopes, three occurred using a high intensity stimulation protocol under pro-epileptogenic medication or following sleep-deprivation and one was only uncertainly related to the stimulation. The risk of rTMS to induce seizures is therefore considered to be very low, furthermore, the new guidelines report that the risk of rTMS-induced seizures does not increase with frequency of stimulations [37]. For iTBS, the risk of inducing seizures is found to be low [53]. Recording of ongoing EEG during rTMS in this study allows monitoring brain activity throughout the experiment and stopping the experiment any time if epileptic brain activity is detected.

In addition to the risk of seizures, TMS-induced headache is a minor risk. The headaches are usually light or moderate in intensity and self-limiting.

Electroencephalography (EEG) and electromyography (EMG), passively record electrical biosignals and have no side-effects; irritation of the skin may be provoked after application of the electrode cream.

The overall risk for subjects participating in this trial therefore can be considered very low.

### Frequency and plans for auditing trial conduct

Complete and correct data collection and storage will be supervised under responsibility of the principal investigator. The study center is affiliated with the Center for Clinical Studies (Zentrum für klinische Studien – ZKS) of the University Hospital Tübingen. This association comes with a yearly external audit as well as regular internal audits (at least two per year) monitoring the accordance of study procedures with the law and internal standard operating procedures following the International Organization of Standardization (ISO) standard 9001.

## Dissemination plans

### Dissemination policy

The results of this study will be disseminated via open-access publications in peer-reviewed journals, and presentations in local, national and international meetings and conferences, exclusively using anonymized data. No other publication restrictions apply.

## Discussion

There is an increasing understanding of the pivotal part played by neural oscillations in network diseases such as MDD, with significant implications for the development of personalized treatment strategies in the evolving landscape of targeted TMS in psychiatric disorders [54]. Major depressive disorder (MDD) continues to represent a leading cause of disability worldwide, and the substantial proportion of patients who fail to respond to conventional treatments highlights the urgent need for novel therapeutic approaches [1, 3]. Non-invasive brain stimulation has emerged as a promising strategy in this regard, but clinical efficacy remains limited by high inter-individual variability [13]. The present study protocol introduces an innovative approach to address this gap by synchronizing TMS to intrinsic neural oscillations in real time. A first randomized controlled trial using this brain state-dependent stimulation approach was initiated in 2023 to test its efficacy in supporting recovery in patients with motor stroke [55].

By targeting theta oscillations over the DMPFC, the trial builds directly on accumulating evidence that aberrant frontal theta activity is a biomarker of treatment resistance in MDD [18, 19]. Previous proof-of-principle studies demonstrated the feasibility of EEG-triggered stimulation in depression [32], but this will be the first randomized clinical trial to evaluate whether a full treatment course of EEG-synchronized, theta-triggered stimulation can be delivered safely and effectively in patients with MDD. In this way, the trial operationalizes a personalized, brain state-dependent intervention that is directly motivated by pathophysiological models of depression and by prior electrophysiological findings. The primary aim of this randomized-controlled clinical trial is to examine the therapeutic efficacy of theta-phase-synchronized transcranial magnetic stimulation (TMS) targeting the left dorsomedial prefrontal cortex (DMPFC) in comparison to an FDA-approved TMS protocol in the indication of pharmaco-resistant MDD.

In addition to advancing the conceptual rationale, the study incorporates several methodological strengths. First, the use of MRI-guided neuronavigation and real-time EEG processing ensures precise targeting of stimulation both spatially and temporally. Second, by including an active comparator arm with FDA-approved iTBS, the design permits a rigorous evaluation of the added value of brain state-dependent timing beyond standard of care. Third, the double-blind parallel-group design, standardized outcome assessments, and predefined statistical plan contribute to transparency and reproducibility. Together, these features align the protocol with current recommendations for clinical trial methodology in neuromodulation research.

Nevertheless, several limitations must be acknowledged. The pilot nature of the trial entails a relatively small sample size, which reduces statistical power and limits the generalizability of the results. Given the exploratory nature of this pilot trial, the study is designed to evaluate the feasibility and potential superiority of DMPFC-EEG-TBS compared to standard iTBS; however, the sample size is insufficient to confirm small or moderate between-group differences, and future adequately powered RCTs will be required. Effect-size estimates will be derived from this study. 

Moreover, the demanding technology of EEG-synchronized TMS, requiring real-time phase detection and neuronavigation, may limit scalability in routine clinical practice at present. Improvements in the practicability of the brain state-dependent stimulation setup will certainly be necessary for clinical use in the long term. First developments in this direction (e.g., application-based operation of the closed-loop device) are currently being made and are seen in a rather straightforward manner to be realized in the near future [54].

A further limitation concerns the choice of oscillatory phase for closed-loop stimulation. While prior phase-specific TMS work has demonstrated that stimulation at the trough of ongoing oscillations yields the most consistent and long-lasting plasticity-like effects [30, 31, 39], direct comparisons between trough-locked and rising/falling-phase stimulation in prefrontal cortex are currently lacking. Thus, although trough-timing is supported by available mechanistic evidence, the present protocol cannot determine whether alternative phase targets might yield equal or even superior therapeutic effects. Future studies should therefore systematically evaluate different theta-phase timings to optimize closed-loop stimulation strategies.

Another limitation is the absence of a sham control condition, which prevents a full separation of specific neuromodulatory effects from non-specific placebo influences; however, the inclusion of two active comparator arms was prioritized to maximize feasibility and clinical relevance in this first therapeutic application. A critical factor hereby is the comparison of two stimulation protocols with different stimulation targets, DLPFC and DMPFC, respectively. The DMPFC was chosen as a target in the theta oscillation-synchronized condition due to the overall high SNR of theta oscillations in healthy subjects [36]. Previous studies have demonstrated that the qualitative derivation of theta oscillations is technically most effective over the DMPFC [56, 57]. Regarding clinical relevance, several studies have shown the DMPFC as a promising stimulation target in patients with MDD [58–60]. The control condition in this trial was not adapted for stimulation of the DMPFC, as doing so would prevent its use as an FDA-approved standard treatment in the comparative control, and would have rather resulted in another experimental condition.

Also, influential factors such as tolerability of the stimulation within the different treatment conditions can be relevant for clinical feasibility and the overall treatment outcome, thus such aspects will be considered with respect to the treatment arm. We expect that rTMS treatment over the DMPFC will be more comfortable than over DLPFC due to the more medial prefrontal target, yet a longer duration of the treatment needs to be endured (15–20 min). TBS over DLPFC might be more uncomfortable than TBS over DMPFC due to the more laterally located target area closer to the temporalis muscle, which might twitch under stimulation. Yet the treatment duration is shorter (3 min). Tolerability and related complaints of the patients, as well as the reasons for drop out of patients will be documented in the CRFs. Sex and gender may influence responsiveness to rTMS, although evidence is mixed. Some studies have suggested higher response rates in women [61, 62], potentially linked to hormonal influences or anatomical factors such as scalp-to-cortex distance, while others have not found significant sex-related differences in clinical outcomes [63]. Given the limited sample size of this pilot trial, we are not powered to formally test gender-specific effects. Nevertheless, we will document and report the gender distribution of participants and conduct exploratory analyses of treatment response by gender. Regarding inter-individual anatomical variability in terms of responsiveness to TMS, in the present protocol the stimulation intensity is defined relative to the resting motor threshold without correction for e.g. scalp-to-cortex distance as we prioritized adherence to the established FDA-approved iTBS standard protocol. Nevertheless, all participants undergo structural MRI, which will enable exploratory analyses of how anatomical factors may relate to treatment outcome. In a subsequent confirmatory trial, we plan to incorporate scalp-to-cortex distance correction or electric-field modeling to improve individual dose standardization.

Finally, stimulation was synchronized to the conventional 4–8 Hz theta band rather than individualized frequency definitions. While this pragmatic approach ensures real-time feasibility, it may not capture inter-individual variability in spectral markers. This approach was chosen to guarantee stable band-pass filtering and reliable phase estimation during stimulation, which is essential for the feasibility of a EEG-synchronized protocol. We are aware that individualized definitions of theta, for example relative to the individual alpha frequency, may in principle provide a more tailored target and have been explored in cognitive neuroscience [64]. However, such procedures are technically challenging to implement in real time. For this reason, we adopted a pragmatic solution for the pilot trial, while planning exploratory offline analyses to examine whether individualized spectral markers might offer added value for guiding stimulation in future confirmatory studies. Although the effective inter-burst repetition rate is slow (~ 0.3 Hz), plasticity is plausibly driven by the gamma-frequency content within each burst nested in theta-phase timing, a canonical gamma–theta phase–amplitude coupling motif implicated in learning [65],in contrast, low-frequency single-pulse rTMS at ~ 0.3 Hz would not be expected to induce LTP-like changes [66].

Despite these constraints, the trial is expected to provide important feasibility and proof-of-concept data. If the results demonstrate that EEG-synchronized stimulation is safe, feasible, and at least non-inferior to standard iTBS, this would justify larger, multi-center trials powered to test clinical efficacy and to refine stimulation parameters further. Positive findings would also pave the way toward more personalized TMS interventions guided by electrophysiological biomarkers, ultimately aiming to improve remission rates in treatment-resistant depression.

In conclusion, this protocol represents an essential step toward translating advances in real-time EEG–TMS integration into clinical practice. By combining established therapeutic stimulation protocols with state-dependent timing, the trial addresses a central limitation of current TMS practice—its lack of personalization. The outcomes of BOSSFRONT2 will inform the design of subsequent confirmatory RCTs and contribute to the development of refined personalized approaches for MDD.

## Supplementary Information

Below is the link to the electronic supplementary material.

Supplementary file1 (PDF 132 KB)

Supplementary file2 (PDF 177 KB)

## Data Availability

Not applicable. This study protocol manuscript does not contain data or materials.

## Abbreviations

ATHF: Antidepressant treatment history form
BDI: Beck’s depression inventory
CRF: Case report form
DMPFC: Dorsomedial prefrontal cortex
DLPFC: Dorsolateral prefrontal cortex
EEG: Electroencephalogram
EMG: Electromyography
HDRS: Hamilton depression rating scale
IKEAB: Institute of clinical epidemiology and applied biostatistics of the University of Tübingen
IDS-30: Inventory of depressive symptom-30
iTBS: Intermittent theta burst stimulation
MADRS: Montgomery–Åsberg depression rating scale
MDD: Major depressive disorder
MEP: Motor-evoked potential
MRI: Magnetic resonance imaging
RMT: Resting motor threshold
rsEEG: Resting state EEG
SNR: Signal-to-noise ratio
TBS: Theta burst stimulation
TMS: Transcranial magnetic stimulation
rTMS: Repetitive TMS
