# Supplementary material for: Brain oscillation-synchronized stimulation for major depression: a randomized controlled trial comparing EEG-triggered repetitive TMS with standard iTBS (Acronym: BOSSFRONT2)
Source: Eur Arch Psychiatry Clin Neurosci. 2026 Jan 21;276(4):1381–98. doi: 10.1007/s00406-025-02176-9 (PMC13233643; doi:10.1007/s00406-025-02176-9)
Supplement: Supplementary file 2 — Supplementary file2 (PDF 177 KB) [file 406_2025_2176_MOESM2_ESM.pdf]

EBERHARD KARLS UNIVERSITÄT TÜBINGEN

Universitätsklinikum Tübingen

Medizinische Fakultät

Ethik-Kommission

Prof. Dr. med. Karl Jaschonek
Vorsitzender

Telefon: +49 7071 29-77661
Telefax: +49 7071 29-5965
E-Mail:
ethik.kommission@med.uni-tuebingen.de

Ethik-Kommission an der Medizinischen Fakultät der Eberhard-Karls-Universität und am Universitätsklinikum Tübingen, Gartenstraße 47, 72074 Tübingen

Frau
Dr. med. Anne Lieb
Neurologische Universitätsklinik
Hoppe-Seyler-Straße 3
72076 Tübingen

nachrichtlich:
Herrn Prof. Dr. med. Andreas Fallgatter, Herrn Prof. Dr. med. Christian Plewnia, Herrn Prof. Dr. med. Ulf Ziemann

**529/2019BO1**	04.11.2022	07.11.2022
unsere Projekt-Nummer	eingegangen am	Datum

Brain oscillation-synchronized stimulation of the frontal cortex: Randomized controlled trial comparing the therapeutic effectiveness of frontal theta-oscillation synchronized repetitive TMS with standard TMS therapy in major depressive disorder. BOSSFRONT2

Hier: Amendment vom 26.10.2022: Anschreiben vom 26.10.2022, Studienprotokoll Version 3 vom 16.09.2022, Probandeninformation vom 16.09.2022

Sehr geehrte Frau Dr. Lieb,

die ergänzenden Unterlagen zur o.g. Studie haben der Ethik-Kommission zur Beratung vorgelegen.
Danach bestehen gegen die Durchführung dieser Studie seitens der Kommission keine Bedenken.

Für die Durchführung Ihres Studienvorhabens wünschen wir viel Erfolg.

Mit freundlichen Grüßen

gezeichnet	für die Richtigkeit<br>i.A.
Prof. Dr. med. Karl Jaschonek<br>Vorsitzender der Ethik-Kommission	Dr. med. Olga Scheck<br>Leiterin der Geschäftsstelle

Digital unterschrieben von Olga Scheck
Datum: 2022.11.07 14:23:42 +01'00'

Seite 2: Mitgliederliste, allgemeine Hinweise

Vorsitz der Ethik-Kommission

Professor Dr. med. Karl Jaschonek	(Vorsitzender)	Innere Medizin
Professor Dr. med. Dr. phil. Urban Wiesing	(1. stellv. Vorsitzender)	Medizinische Ethik
Professor Dr. med. Dieter Luft	(2. stellv. Vorsitzender)	Innere Medizin

Mitglieder der Ethik-Kommission

Professor Dr. med. Berthold Drexler	Anästhesiologie
Professor Dr. med. Jürgen Honegger	Neurochirurgie
Professor Dr. med. dent. Bernd Koos	Zahnmedizin
Professor Dr. phil. Dipl. Psych. Stefan Klingberg	Psychologie, Psychotherapie
Professor Dr. med. Holger Lerche	Neurologie
Professor Dr. rer.nat. Peter Martus	Biometrie
Professor Dr. med. Klaus Mörike	Klinische Pharmakologie
Professor Dr. med. Christian F. Poets	Pädiatrie
Ulrike Röllecke	Laie
Professor Dr. iur. Dr. h. c. Georg Sandberger	Jurist
Dipl.-Ing. Frank Stegmaier	Medizinproduktesicherheit

Die Ethik-Kommission an der Medizinischen Fakultät der Eberhard-Karls-Universität und am Universitätsklinikum Tübingen verfährt entsprechend den ICH-GCP-Richtlinien, der Deklaration von Helsinki in der jeweils gültigen Fassung sowie den gesetzlichen Bestimmungen. Die Ethik-Kommission ist bei den Bundesoberbehörden registriert.

Die Ethik-Kommission bestätigt, dass der Prüfplan mit den erforderlichen Unterlagen insbesondere nach ethischen und rechtlichen Gesichtspunkten beraten wurde. Die berufsethische und berufsrechtliche Beratung gemäß §15 Abs.1 Berufsordnung für Ärzte in Baden-Württemberg ist für 3 Jahre ab Ausstellungsdatum gültig.
Die Ethik-Kommission bestätigt, dass der Prüfplan mit den erforderlichen Unterlagen, insbesondere nach ethischen und rechtlichen Gesichtspunkten, mündlich beraten wurde. Die berufsethische und berufsrechtliche Beratung gemäß §15 Abs.1 Berufsordnung für Ärzte in Baden-Württemberg ist für 3 Jahre ab Ausstellungsdatum gültig.
Änderungen im Prüfplan und in der Phase der Umsetzung bitten wir der Kommission mitzuteilen; dabei wären wir Ihnen dankbar, wenn Sie geänderte Passagen deutlich kennzeichnen würden.
Unabhängig vom Beratungsergebnis macht die Ethik-Kommission darauf aufmerksam, dass die medizinische, ethische und rechtliche Verantwortung für die Durchführung des Forschungsvorhabens beim Projektleiter und allen an der Studie teilnehmenden Ärzten liegt.
Datenschutzrechtliche Aspekte des Forschungsvorhabens wurden nur kursorisch geprüft. Das Votum der Ethik-Kommission ersetzt nicht die Konsultation der/des zuständigen Datenschutzbeauftragten. Die Einhaltung der gesetzlichen Vorgaben sowie die Umsetzung des Datenschutzkonzepts liegen in der Verantwortung der Studienverantwortlichen.

Nach Abschluss der Studie bittet die Kommission um einen abschließenden Bericht.
