# Supplementary material for: Brain oscillation-synchronized stimulation for major depression: a randomized controlled trial comparing EEG-triggered repetitive TMS with standard iTBS (Acronym: BOSSFRONT2)
Source: Eur Arch Psychiatry Clin Neurosci. 2026 Jan 21;276(4):1381–98. doi: 10.1007/s00406-025-02176-9 (PMC13233643; doi:10.1007/s00406-025-02176-9)
Supplement: Supplementary file 1 — Supplementary file1 (PDF 132 KB) [file 406_2025_2176_MOESM1_ESM.pdf]

EBERHARD KARLS UNIVERSITÄT TÜBINGEN

Universitätsklinikum Tübingen

Faculty of Medicine

Ethics Committee

Prof. Dr. med. Karl Jaschonek
Chairman

Phone: +49 7071 29-77661
Fax: +49 7071 29-5965
E-Mail:
ethik.kommission@med.uni-tuebingen.de

Ethics Committee at the Medical Faculty of the Eberhard Karls University and at the University Hospital Tübingen, Gartenstraße 47, 72074 Tübingen

Woman
Dr. med. Anne Lieb
Neurological University Hospital
Hoppe-Seyler-Straße 3
72076 Tuebingen

for information:
Prof. Dr. med. Andreas Fallgatter, Prof. Dr. med. Christian Plewnia, Prof. Dr. med. Ulf Ziemann

**529/2019BO1**	04.11.2022	07.11.2022
our project number	received on	Date

Brain oscillation-synchronized stimulation of the frontal cortex: Randomized controlled trial comparing the therapeutic effectiveness of frontal theta-oscillation synchronized repetitive TMS with standard TMS therapy in major depressive disorder. BOSSFRONT2

Here: Amendment dated 26.10.2022: Cover letter dated 26.10.2022, study protocol version 3 dated 16.09.2022, subject information dated 16.09.2022

Dear Dr. Lieb,

The supplementary documents for the above-mentioned study have been submitted to the Ethics Committee for consultation.
Accordingly, the Commission has no objections to the implementation of this study.

We wish you every success with your study project. Yours sincerely

drawn

Prof. Dr. med. Karl Jaschonek
Chairman of the Ethics Committee

for the correctness
on behalf of
Dr. med. Olga Scheck
Head of the office

Digitally signed
by Olga Scheck
Date: 2022.11.07
14:23:42 +01'00'

Page 2: List of members, general information

University Hospital Tübingen
Institution under public law
Headquarters Tübingen
Geissweg 3 - 72076 Tübingen
Tel. 07071/29-0
www.medizin.uni-tuebingen.de
Tax no. 86156/09402
VAT ID: DE 146 889 674

Supervisory Board
Dr. Hans J. Reiter (Chairman)
Management Board
Prof. Dr. Michael Bamberg (Chairman)
Gabriele Sonntag (Deputy Chairwoman)
Prof. Dr. Karl Ulrich Bartz-Schmidt
Prof. Dr. Bernd Pichler
Klaus Tischler

Baden-Württembergische Bank Stuttgart
BLZ 600 501 01 Account no. 7477 5037 93
IBAN: DE 41 6005 0101 7477 5037 93
BIC (SWIFT code): SOLADEST600
District Savings Bank Tübingen
Sort code 641 500 20 Account no. 14 144
IBAN: DE 79 6415 0020 0000 0141 44
BIC (SWIFT code): SOLADES1TUB

Chair of the Ethics Committee

Professor Dr. med. Karl Jaschonek	(Chairman)	Internal medicine
Professor Dr. med. Dr. phil. Urban Wiesing	(1st Deputy Chairman)	Medical ethics
Professor Dr. med. Dieter Luft	(2nd Deputy Chairman)	Internal medicine

Members of the Ethics Committee

Professor Dr. med. Berthold Drexler	Anesthesiology
Professor Dr. med. Jürgen Honegger	Neurosurgery
Professor Dr. med. dent. Bernd Koos	Dentistry
Professor Dr. phil. Dipl. Psych. Stefan Klingberg	Psychology, psychotherapy
Professor Dr. med. Holger Lerche	Neurology
Professor Dr. rer.nat. Peter Martus	Biometrics
Professor Dr. med. Klaus Mörike	Clinical pharmacology
Professor Dr. med. Christian F. Poets	Pediatrics
Ulrike Röllecke	layman
Professor Dr. iur. Dr. h. c. Georg Sandberger	Medical device safety lawyer
Dipl.-Ing. Frank Stegmaier	

The Ethics Committee at the Medical Faculty of the Eberhard Karls University and the University Hospital Tübingen acts in accordance with the ICH-GCP guidelines, the Declaration of Helsinki as amended and the statutory provisions. The Ethics Committee is registered with the higher federal authorities.

The Ethics Committee confirms that the protocol with the required documents has been discussed in particular from an ethical and legal point of view. The ethical and legal advice in accordance with §15 para. 1 of the Professional Code for Physicians in Baden-Württemberg is valid for 3 years from the date of issue.
The Ethics Committee confirms that the protocol has been discussed orally with the necessary documents, in particular with regard to ethical and legal aspects. The ethical and legal advice in accordance with
§15 Abs.1 Berufsordnung für Ärzte in Baden-Württemberg is valid for 3 years from the date of issue.
Please inform the Commission of any changes in the audit plan and in the implementation phase; we would be grateful if you would clearly mark any amended passages.
Irrespective of the outcome of the consultation, the Ethics Committee would like to point out that the medical, ethical and legal responsibility for the implementation of the research project lies with the project leader and all doctors participating in the study.
Data protection aspects of the research project were only examined cursorily. The vote of the Ethics Committee does not replace the consultation of the responsible data protection officer. Compliance with the legal requirements and the implementation of the data protection concept are the responsibility of those responsible for the study.

Once the study has been completed, the Commission will request a final report.
